# Insight into the dynamics of heat and mass transfer in nanofluid flow with linear/nonlinear mixed convection, thermal radiation, and activation energy effects over the rotating disk

**DOI:** 10.1038/s41598-023-49988-0

**Published:** 2023-12-27

**Authors:** Shumaila Kanwal, Syed Asif Ali Shah, Abdul Bariq, Bagh Ali, Adham E. Ragab, Emad A. Az-Zo’bi

**Affiliations:** 1 Department of Mathematics and Statistics, The University of Lahore, Lahore, Pakistan; 2 Department of Mathematics, Leghman University, Mehtarlam, Laghman 2701 Afghanistan; 3 School of Mechanical Engineering and Automation, Harbin Institute of Technology, Shenzhen, 518055 China; 4 Department of Industrial Engineering, College of Engineering, King Saud University, P.O. Box 800, 11421 Riyadh, Saudi Arabia; 5 Department of Mathematics, Mutah University, Mutah, Al Karak Jordan

**Keywords:** Engineering, Mathematics and computing

## Abstract

In this paper, we study linear and nonlinear mixed convection, activation energy, and heat radiation effects caused by nanoparticles. This study aims to improve the understanding of how nanofluids behave in the presence of rotating disks and develop more efficient and effective cooling technologies. The flow problem consisted of partial differential equations (PDE). It is challenging to calculate these equations as a result of these nonlinear PDEs. Consequently, we use appropriate similarities to transform them into ordinary differential equations (ODEs). The bvp4c Matlab built-in technique is then used to resolve these ODEs. The velocities, temperature, and concentration outcomes with the various factors are examined graphically. Additionally, tables are employed to analyze the skin friction and Nusselt number values. It is analyzed that increasing the linear and linear mixed convection parameters enhances the velocity profiles of nanofluid. Enhancements in heat are analyzed by increasing nonlinear thermal radiation and enhancement in concentration is examined by increasing activation energy. Furthermore, as the variables for thermophoresis and Brownian motion are increased, the Nusselt number falls. The heat transfer rate is 27.16% for $Rd=0.6$ and 39.28% for $Rd=1.4$. Thus, the heat transfer rate is enhanced 12.12%. This study’s practical applications include improving the behavior of fluids and the transfer of heat in rotating frameworks, which may affect energy systems, heat exchangers, and cooling advances in technology.

## Introduction

Nanofluid is an interruption of nanoparticles (at least one dimension less than 100 nm). Oil recovery, medical devices, the textile sector, and solar heating applications using nanofluids to improve heat transfer. Nanofluids in a base-fluid have a high value of thermal conductivity. It also has coefficients in a single phase, wettability, and rheological properties, which will enhance the performance of different applications i.e. heat transfer, drug delivery enhanced oil recovery and lubrication, etc. The mono nanofluids have just one type of nanoparticle. It discloses the fixed little thermal properties. There are a few ways that nanofluids improve heat and mass transfer such as thermal conductivity, convective heat transfer, surface area, nanoparticle concentration, shape and size, etc. Choi and Eastman^1^ first used the term “nanofluid” to describe a special category of heat-transfer fluids based on nanotechnology that has better thermal characteristics than their base fluids or ordinary particle-fluid suspensions. Turkyilmazoglu^2^ studied about nanofluid flow boundary layer above a spinning disk. In this paper, the flow and temperature fields, together with the shear stress and heat exchange parameters, were calculated for specific values of nanoparticle volume fraction. The characteristics of heat transmission in nanofluids were discussed by Ganvir et al.^3^. They discussed the impact of temperature, usage of surfactants to improve the steadiness of nanoparticles, convection of heat transmission, thermal properties, and size of particles effects. The amount of heat transfer on the surface was calculated using the coefficient of thermal conductivity and the thickness of linked layers. Lenin et al.^4^ discussed how thermal properties are affected by incorporating layer thickness. They concluded that their research supported thermal properties’ usefulness and practical application, which generates revenue. In the context of both base materials and a field of magnets, Ali et al.^5^ examined the importance of variable nanomaterial radius for the non-Newtonian flow of nanofluid induced by an expanding sheet. Khan et al.^6^ investigate Sutterby nanofluid flow with nonlinear thermal radiation. They observed that the concentration profile declines for higher inputs of Brownian motion parameters. Khan^7,8^ studied different non-Newtonian nanofluid flows for various geometries. In these models, he analyzed that heat transmission is enhanced in nanofluids. The flow of a combination of two particles called hybrid in fluid through a rotating sphere with heat radiation was examined by Ramesh et al.^9^.

Physical systems’ behavior and characteristics are greatly influenced by boundary conditions. They specify the restrictions placed on a system at its limits, and they are important physically for several reasons such as system stability and Well-posedness, system characterization, and conservation laws. The slip boundary impact of a rough rotating disk flow was investigated by Wang^10^ and Hayata et al.^11^. Huminic and Huminic^12^ discussed various boundary values and the presence of various physical situations in the different thermal systems created the entropy of hybrid nanofluid and nanofluid. Alsallami et al.^13^ Studied the numerous facets of the heat and mass transition in terms of Arrhenius activation energy. Ali et al.^14^ studied the MHD not constant transfer of spinning Maxwell flow of nanofluid where Arrhenius activation energy was used to evaluate the movement of chemically bonded species. Nanofluids are being used increasingly in profitable and definite technological parts of the business including lubricating systems, cancer treatment, antibacterial technologies, and solar cell improvement. Shahid et al.^15^ examined the viscosity factor of magnetohydrodynamics (MHD) nanofluid, which depends on temperature. They analyzed that the transportation of heat increased when the force of transportation increased and the transportation of heat decreased when the Brownian motion variable increased. Alshbool et al.^16^ investigated the numerical MHD flow of activation energy with variable viscosity dependent on temperature. Along with all the current factors for temperature, velocity, and concentration distributions, they also addressed skin friction and Nusselt number. The effects of MHD, heat radiation, and activation energy in the micropolar nanofluid flow were examined by Alanazi et al.^17^. They analyzed through the use of the shooting approach, systematic answers were produced and considered appropriately. It has been observed that distributions of application and temperature are raised by greater levels of thermophoretic variables. Jamshed et al.^18^ investigated the flow of MHD nanofluid by the transportation of heat. It is very important to keep in mind that the magnetic field exists here then it will raise both the non-constant temperature and the arrangement of nanoparticles. Khan et al.^19,20^ examined more results about the flow of a nanofluid within the existence of mass and heat radiation.

When free/natural convection and forced convection techniques synergistically collaborate to facilitate heat transfer, this phenomenon is recognized as mixed convection within the respective field. Ali et al.^21^ explored the improved thermal transfer for mixed Casson flow of nanofluid with a magnetic field across a spinning cylinder. Safdar et al.^22^ studied mixed-convection Maxwell nanofluid flow with the effects of MHD and thermal radiation across the porous medium. Electromagnetic radiation that a substance emits as a result of its heat, and whose properties depend on the material’s temperature. Shiekholeslami et al.^23^ studied experimentally the MHD flow of nanofluid with heat radiation. They noticed that as the heat parameter value boosted the temperature profiles decreased. Younis et al.^24^ examined nanofluid flow over a cavity with the affect on MHD mixed convection and heat radiation. Wakif et al.^25^ considered the model of nanofluid in one and two-dimensional by the presence of heat radiation. Song et al.^26^ examined the application of the flow of nanofluid above the spinning disk. Qureshi^27^ discussed the numerical solution of nanofluid in the presence of heat radiation.

The least possible energy was observed in the study of the kinetics phase in solid and liquid. This can happen as a nonlinear holding in the middle of activation energy and conversion. Activation energy is a multi-stage process. It is specified with a mutual parameter which is joined with the individual steps of activation energies. This association can be constructed in algebraic form by employing proper models of the processes. So, through this, we can study the presence of activation energy in the experiment. We can determine the different steps of activation energy. We can also evaluate the different parameters of the process acting on the experiment. It offers numerous polymer melting and transition processes of glass. Additionally emphasized is the usage of proper computational methods. The benefits and drawbacks of using the activation energy to assess thresholds, excitation functions, and in the resistance of tunneling processes. Khan et al.^28^ discussed the activation energy in MHD flow close to the stagnation area in the direction of the stretching surface. Their study’s primary research focuses were the characterization of velocity, heat, and nanoparticle concentration. The investigation of shape effects in nanofluids was crucial for both fluid flow and heat transfer.

Kotresh et al.^29^ noted that higher solid volume percent values increase the drag coefficient and slow down the rate of heat transmission. Raju et al.^30^ analyzed cross-diffusion effects and hybrid nanofluid flow between rotary disks with unsustainability. Asma et al.^31^ investigated three-dimensional MHD nanofluid flow caused by a revolving disk exposed to heat generation and absorption. They examined a novel study of nonlinear convective flow in the Sisko model on a rotating disk that can be stretched horizontally in the presence of a steady vertical field. They also investigated the optimization of the amount of entropy and activation energy. In addition, consideration of the non-uniform heat and radiation absorption, MHD, viscous dissipation, and Joule heating was reviewed by^32,33^. The transfer of heat over the rotating disk in the presence of a thermal effect was studied by Khan et al. and Basha and Sivaraj^34,35^. They collected the improvements in convection variables. Basha et al.^36^ employ the present model to investigate the radiative nano-magnetic insulating flow. Ullah et al.^37^ Studied the flow of nanofluid and the effects of activation energy on it. From the above literature, it is analyzed that there is no study on heat and mass transfer in nanofluid flow. This puts us in a position to study the nanofluid flow across the rotating disk. In this research, we study the nanofluid flow over the rotating disk and the novelties of this study are:

(i) The effects of linear and nonlinear mixed convection, and MHD are analyzed
(ii) The effect of nonlinear radiation is taken into account.
(iii) The impact of activation energy is analyzed.
(iv) Buongiorno model is considered.
(v) The slip boundary conditions are analyzed.

## Mathematical formulation

**Figure 1:**
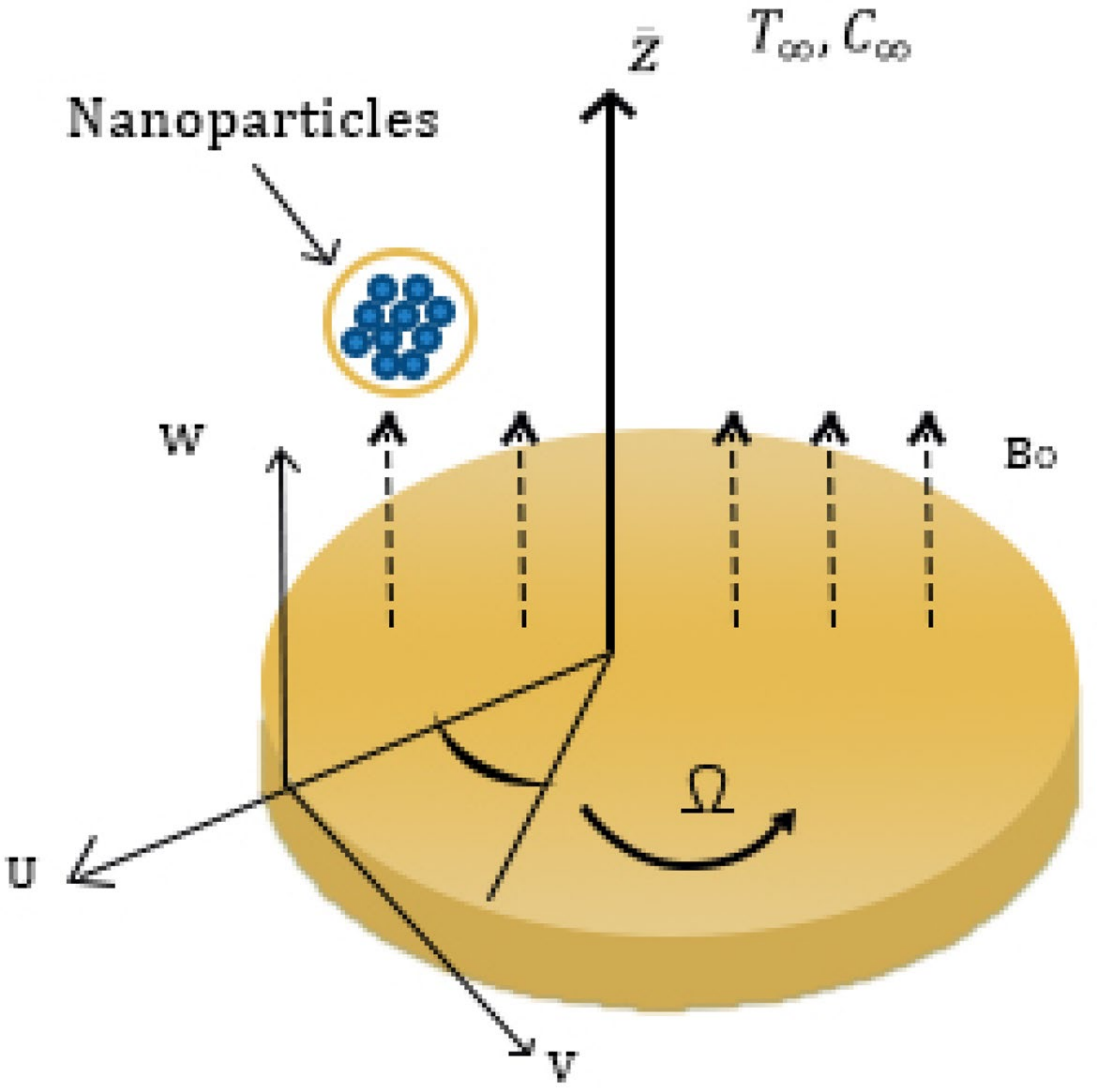
Geometrical shape of the nanoparticles flow.

Suppose the nanoparticle flows across an infinitely large rough disc, which is placed in the z-plane ${\bar{z}}=0$. The investigation has chosen to use the $({\bar{r}},\varphi ,{\bar{z}})$ spherical coordinates. An unsteady, electrically carrying nanofluid fills the half-filled space ${\bar{z}}$ is greater than 0. Consequently, the disc rotates at a uniform angular velocity of ${\bar{\Omega }}$, leading to the flow being generated towards the positive $\varphi$. Partial slip conditions are assumed to be relevant as the fundamental level of unevenness at the disk is believed to be smaller than the distance between boundary layers. A magnetic field with constant strength $(B_{o})$, operates axially. As the magnetic Reynolds number is so small, it is expected that the magnetic field will be neglected. Let $(T_w)$ and $(T_{\infty })$ stand for the wall and atmospheric temperatures, respectively, where $T_w$ is greater than $T_{\infty }$. The coordinate-respective derivatives $\varphi$ are eliminated due to the original problem of inversion symmetry as shown in Fig. 1.

The Buongiorno structure can be employed to convert the equation system to sequential approaches^34,38,39^.

1 $$\begin{aligned}{} & {} \frac{\partial U}{\partial {\bar{r}}}+\frac{\partial W}{\partial {\bar{z}}}+\frac{U}{{\bar{r}}}=0, \end{aligned}$$

2 $$\begin{aligned}{} & {} \rho _{f}\left( \frac{\partial U}{\partial {\bar{r}}} U + \frac{\partial U}{\partial {\bar{z}}} W -\frac{V^2}{{\bar{r}}}\right) =-\frac{\partial {\bar{p}}}{\partial {\bar{r}}}+\mu _f\left( \frac{\partial ^2 U}{\partial \bar{r^2}}+\frac{\partial ^2 U}{\partial \bar{z^2}}+\frac{1}{{\bar{r}}}\frac{\partial U}{\partial {\bar{r}}}- \frac{U}{\bar{r^2}}\right) -\sigma ^{*}_{f} B_{0}^{2} U \nonumber \\{} & {} \quad + g^{*}\lambda _{1} ({\bar{C}} - C_{\infty })+ g^{*} \lambda _{2} ({\bar{C}}-C_{\infty })^{2}+g^{*} \lambda _{3} ({\bar{T}}-T_{\infty })+ g^{*}\lambda _{4} ({\bar{T}}-T_{\infty })^{2}, \end{aligned}$$

3 $$\begin{aligned}{} & {} \rho _{f}\left( \frac{\partial V}{\partial {\bar{r}}} U+\frac{U V}{{\bar{r}}}+ \frac{\partial V}{\partial {\bar{z}}} W\right) =\mu _f\left( \frac{\partial ^2 V}{\partial \bar{r^2}}+\frac{1}{{\bar{r}}}\frac{\partial V}{\partial {\bar{r}}}- \frac{V}{\bar{r^2}}+ \frac{\partial ^2 V}{\partial \bar{z^2}}\right) -\sigma ^{*}_{f}B_{0}^{2} V \nonumber \\{} & {} \quad +g^{*}\lambda _{1} ({\bar{C}} - C_{\infty })+ g^{*} \lambda _{2} ({\bar{C}}-C_{\infty })^{2}+g^{*} \lambda _{3} ({\bar{T}}-T_{\infty })+ g^{*}\lambda _{4} ({\bar{T}}-T_{\infty })^{2}, \end{aligned}$$

4 $$\begin{aligned}{} & {} \rho _{f}\left( \frac{\partial W}{\partial {\bar{r}}}U+\frac{\partial W}{\partial {\bar{z}}} W \right) =\mu _f\left( \frac{\partial ^2 W}{\partial \bar{r^2}}+\frac{\partial ^2 W}{\partial \bar{z^2}}+\frac{1}{{\bar{r}}}\frac{\partial W}{\partial {\bar{r}}}\right) -\frac{\partial {\bar{p}}}{\partial {\bar{z}}}, \end{aligned}$$

5 $$\begin{aligned}{} & {} \frac{\partial {\bar{T}}}{\partial {\bar{r}}} U+ \frac{\partial {\bar{T}}}{\partial {\bar{z}}}W={\bar{\tau }} {{{\bar{D}}}_B} \left( \frac{\partial {\bar{T}}}{\partial {\bar{r}}}\frac{\partial {\bar{C}}}{\partial {\bar{r}}}+\frac{\partial {\bar{T}}}{\partial {\bar{z}}} \frac{\partial {\bar{C}}}{\partial {\bar{z}}}\right) +{\bar{\tau }} \frac{{{\bar{D}}}_T}{T_{\infty }} \left( \frac{\partial {\bar{T}}}{\partial {\bar{r}}}\right) ^2 +{\bar{\tau }} \frac{{{\bar{D}}}_T}{T_{\infty }} \left( \frac{\partial {\bar{T}}}{\partial {\bar{z}}}\right) ^2 \nonumber \\+{} & {} \quad \alpha _f \left( \frac{\partial ^{2} {\bar{T}}}{\partial \bar{r^{2}}}+\frac{1}{{\bar{r}}}\frac{\partial {\bar{T}}}{\partial {\bar{r}}} + \frac{\partial ^{2} {\bar{T}}}{\partial \bar{z^{2}}}\right) +\frac{16\sigma _1 T_{\infty }^3}{3\left( \rho c \right) _f K} \,\,\ \frac{\partial }{\partial {\bar{z}}}\left( \bar{T^{3}}{\frac{\partial {\bar{T}}}{\partial {\bar{z}}}}\right) , \end{aligned}$$

6 $$\begin{aligned}{} & {} \frac{\partial {\bar{C}}}{\partial {\bar{r}}}U+ \frac{\partial {\bar{C}}}{\partial {\bar{z}}} W =\frac{{{\bar{D}}}_T}{T_{\infty }} \left( \frac{\partial ^2 {\bar{T}}}{\partial \bar{r^2}} +\frac{\partial ^{2}{\bar{T}}}{\partial {\bar{z}}^{2}}+\frac{1}{{\bar{r}}} \frac{\partial {\bar{T}}}{\partial {\bar{r}}} \right) +\bar{D_B}\left( \frac{\partial ^2 {\bar{C}}}{\partial \bar{r^2}}+\frac{1}{{\bar{r}}}\frac{\partial {\bar{C}}}{\partial {\bar{r}}} + \frac{\partial ^2 {\bar{C}}}{\partial \bar{z^2}}\right) \nonumber \\{} & {} \quad -K_R^{2} \left( {\bar{C}}- C_\infty \right) \left( \frac{{\bar{T}}}{T_{\infty }}\right) ^n exp\left( \frac{-E^{*}}{K T}\right) . \end{aligned}$$

Here *W*, *V* and *U* stand for velocity components in the orientation with rising ${\bar{z}}$, $\phi$, and ${\bar{r}}$ respectively. ${\bar{C}}$ denotes the nanoparticles concentration, ${\bar{p}}$ indicates pressure, $\rho _f$ stand for base fluid density, ${\bar{T}}$ denotes the local temperature fluid’s, $\nu _f$ represents the kinematic viscosity of the base fluid, $\lambda _1$ and $\lambda _2$ are mixed convection due to concentration, $\lambda _3$, $\alpha _f$ indicates the thermal diffusivity of the nanofluid and $\lambda _4$ are mixed convection due to temperature, $g^{*}$ is gravitational acceleration, $K_{R}$ chemical reaction factor, The letters $\bar{D_B}$, ${{\bar{\tau }}}$ and $\bar{D_T}$ indicate the Brownian diffusion coefficient, the value of the capacity of the heat of base liquid to the material of nanoparticle and thermal diffusion rate, respectively.

For the problem, these limitations are applicable^40^.

7 $$\begin{aligned} \text {when} ~{\bar{z}}= & {} 0, \,\,\,\,\ V=\bar{L_1}\frac{\partial V}{\partial {\bar{z}}}+{\bar{r}} {\bar{\Omega }}, \,\,\,\,\ W=0, \,\,\,\,\ U =\bar{L_1}\frac{\partial U}{\partial {\bar{z}}}, \,\,\,\,\ \nonumber \\ {\bar{T}}= & {} T_{w}+L_2\frac{\partial {\bar{T}}}{\partial {\bar{z}}}, \,\,\,\,\ \nonumber \\ {\bar{C}}= & {} C_{w}+L_2\frac{\partial {\bar{C}}}{\partial {\bar{z}}}=0, \nonumber \\{} & {} \text {when} ~{\bar{z}}\rightarrow \infty , \,\,\,\,\ ~{\bar{C}} \rightarrow C_{\infty }, \,\,\,\,\ {\bar{T}} \rightarrow T_{\infty }, \,\,\,\,\ ~{\bar{p}} \rightarrow p_{\infty }, \,\,\,\,\ ~U \rightarrow 0. \end{aligned}$$

In this case, ${\bar{L}}_1$ indicates the wall slip ratio and ${\bar{L}}_2$ refers to the increasing ratio of temperature.

The approximate solution provided here can be used to convert the resulting PDEs into ODEs^11,41^;

8 $$\begin{aligned} U=\, & {} f'\left( \eta \right) r {\bar{\Omega }}, \,\,\,\,\,\ W = - f\left( \eta \right) \sqrt{2 \nu _{f} {\bar{\Omega }}}, \,\,\,\,\,\ V = r {\bar{\Omega }} g \left( \eta \right) , \,\,\,\,\,\ \eta = {\bar{z}} \sqrt{\frac{2 {\bar{\Omega }}}{\nu _{f}}} \nonumber \\ {\bar{C}}=\, & {} \phi \left( \eta \right) C_{\infty } + C_{\infty }, \,\,\,\,\,\ {\bar{p}}=p_{\infty }-\mu _f {\bar{\Omega }} P\left( \eta \right) , \,\,\ {\bar{T}}= \theta \left( \eta \right) \left( T_{w} - T_{\infty } \right) +T_{\infty }. \end{aligned}$$

For all the non-dimensional parameters *f*, *g*, $\theta$, and $\phi$, $\eta$ is the non-dimensional length along the rotational axis. Equations (1) and (3) have comparable justifications when using the similarity transformation ratio from the preceding equations. We reorganize Eqs (2,3,5,6) as follows;

9 $$\begin{aligned}{} & {} f'''+f''f-\frac{1}{2}f'^{2}-Mf'+\frac{1}{2}g^{2}+\chi _C\left( 1+\beta _C \phi \right) \phi +\chi _T\left( 1+\beta _T \theta \right) \theta =0, \end{aligned}$$

10 $$\begin{aligned}{} & {} g''+ g' f -g f' - M g +\chi _C\left( 1+\beta _C \phi \right) \phi +\chi _T\left( 1+\beta _T \theta \right) \theta =0, \end{aligned}$$

11 $$\begin{aligned}{} & {} 4Rd\left( 1+\left( \theta _w-1\right) \theta \right) ^{2} \left( \theta _w-1\right) \theta {'}^{2}+ \left[ \frac{4}{3}Rd\left( 1+\left( \theta _w-1\right) \theta \right) ^{3}+1\right] \theta ''\nonumber \\{} & {} \quad +\left( Nt\theta '^{2}+f\theta '+Nb\phi '\theta '\right) P_r =0, \end{aligned}$$

12 $$\begin{aligned}{} & {} { \frac{Nt}{Nb}\theta ''+Sc f\phi '+\phi ''-\sigma ^{*} Sc \left( 1+{\hat{\delta }}\theta \right) ^{n}\phi \,\,\ exp \left( \frac{-E}{1+\delta \theta }\right) =0. } \end{aligned}$$

The modified boundary conditions are as follows:

$$\begin{aligned}{} & {} \text {at} ~\eta =0, \,\, \,\ f^{'}=\alpha _1 f^{''}, \,\, \,\, g=1, \,\,\,\, \phi = 1 + \alpha _2\phi ^{'}, \,\, \,\, f =0, \,\, \,\, \theta =1 \,\ \\{} & {} \quad t{at}\,\, ~\eta \rightarrow \infty , \,\,\,\, {\bar{P}}\rightarrow 0, \,\,\,\, f'\rightarrow 0,\,\,\,\, \phi \rightarrow 0, \,\,\,\, \theta \rightarrow 0, \,\,\,\, g\rightarrow 0. \end{aligned}$$

In this case, $f^{'}$ and $g^{'}$ are the prime derivatives with respect to $\eta$, $\alpha _1=\bar{L_1} \sqrt{\frac{2{\bar{\Omega }}}{\nu _{f}}}$ is velocity slip parameter, $\alpha _2=\bar{L_2}\sqrt{\frac{2{\bar{\Omega }}}{\nu _{f}}}$ indicate for thermal slip parameter, $P_r =\frac{\nu _f}{\alpha _f}$ stands for the Prandtl number, $Sc=\frac{\nu _f}{\bar{D_B}}$ is the Schmidt number, $\alpha _f = \frac{k_f}{\left( \rho c \right) _f}$ denotes thermal diffusivity of nanofluid, $N_b =\frac{{\bar{\tau }} \bar{D_B} C_{\infty }}{\nu _f}$ stands for the Brownian motion variable, $\beta _C=\frac{\lambda _2}{\lambda _1}\left( C_\infty \right)$ is quadratic convection variable due to concentration, ${N_t} =\frac{{\bar{\tau }} \bar{D_T} \left( T_w - T_{\infty }\right) }{\nu _f}$ indicates the thermophoresis variable, $\beta _T=\frac{\lambda _4}{\lambda _3}\left( T_w-T_\infty \right)$ is quadratic convection parameter due to tempreture, $\delta = \left( \frac{T_w }{T}-1 \right)$ is heat absorption/generation variable, $\chi _C=\frac{g^{*} \lambda _1 C_{\infty }}{2r{\bar{\omega }}^{2}\rho _f}$ shows a nonlinear mixed convection parameter due to concentration, $E = \frac{E^{*}}{KT_\infty }$ is activation energy variable coefficient, $\chi _T=\frac{g^{*} \lambda _3 \left( T-T_{\infty )}\right) }{2r{\bar{\omega }}^{2}\rho _f}$ nominates a nonlinear mixed convection parameter due to temperature, $Rd= \frac{4\sigma _1 T^{3}_\infty }{KK_f}$ designates thermal radiation, $\theta _w = \frac{T_w}{T_\infty }$ is temperature difference parameter, and $\sigma ^{*}=\frac{Kr^{2}}{2\Omega }$ is chemical reaction parameter.

Take into account that integrating Eq. (4) makes it simple to determine nanofluid pressure. According to the definite integral, the value that represents a practical issue is the frictional force acting on a disc with a radius *R* in this specific case.

13 $$\begin{aligned} {\bar{T}}= -2 \pi r^{2} \int _{0}^{R} \mu _f\frac{\partial V}{\partial {\bar{z}}}\mid _{z=0} = -\frac{\pi \rho _f {\bar{\Omega }}}{2}\sqrt{2\nu _f {\bar{\Omega }} R^{4}} g^{'}\left( 0\right) \end{aligned}$$

Additionally, tangential stress $\hat{\tau _r}$ and radial stress $\hat{\tau _\theta }$ are calculated as:

14 $$\begin{aligned} \bar{\tau _r}= & {} \mu _f\left( \frac{\partial U}{\partial {\bar{z}}}+\frac{\partial W}{\partial {\bar{r}}}\right) _{z=0} = r{\bar{\Omega }} \mu _f \sqrt{\frac{2 {\bar{\Omega }}}{\nu _f}}f^{''}\left( 0\right) , \end{aligned}$$

15 $$\begin{aligned} \bar{\tau _\theta }=\, & {} \mu _f\left( \frac{\partial V}{\partial {\bar{z}}}+\frac{\partial W}{\partial {\bar{r}}}\right) _{z=0} = r{\bar{\Omega }} \mu _f \sqrt{\frac{2 {\bar{\Omega }}}{\nu _f}}g^{'}\left( 0\right) , \end{aligned}$$

which generates the skin friction coefficient that follows;

16 $$\begin{aligned} \bar{C_f}=\frac{\sqrt{{\bar{\tau }}^{2}_r + {\bar{\tau }}^{2}_\theta }}{\rho _f r^{2} {\bar{\Omega }}}^{2} = \sqrt{\frac{\nu _f}{2 {\bar{\Omega }} r^{2}}} \,\,\,\ \sqrt{f^{''}\left( 0\right) ^{2}+g^{'}\left( 0\right) ^{2}}. \end{aligned}$$

We include the heat fluxes caused by the diffusion and conduction of nanoparticles to determine the local Nusselt number ${\bar{Nu}}_r$,

17 $$\begin{aligned} {{\bar{Nu}}_r=\frac{r}{T_w - T_\infty }\left( \frac{\partial {\bar{T}}}{\partial {\bar{z}}}+\frac{16\sigma _1 T_{\infty }^3}{3K_{f} K}\frac{\partial {\bar{T}}}{\partial {\bar{z}}}\right) _{z=0}.} \end{aligned}$$

Consider that the zero wall mass flux supposition prevents the wall heat flux arising from nanoparticle diffusion. Given Eqs. (8, 9), Eq. (19) has the form

18 $$\begin{aligned} {{\bar{Nu}}_r=-r\sqrt{\frac{2{\bar{\Omega }}}{\nu _f}}\left( 1+\frac{4}{3}Rd\right) \theta ^{'}\left( 0\right) .} \end{aligned}$$

## Numerical techniques

The equations relating to the transport of mass, heat, and momentum can be solved using a variety of numerical solvers such as finite difference method (FDM), finite volume method (FVM), finite element method (FEM), boundary element method (BEM), discrete element method (DEM), and boundary value problem 4th order accurate (Bvp4c).

## Bvp4c technique

Here, a direct discretization method and a shooting strategy for tackling boundary value issues have been devised. To solve generic, nonlinear boundary value issues, one would prefer to employ a high-order method that is reliable and capable of doing so. MATLAB’s bvp4c method is a useful and easy-to-use tool that can handle a variety of relatively complex problems. $10^{-6}$ is the convergence rate of Bvp4c. To resolve nonlinear systems of equations, the technique employs an iteration structure. Specifically, the finite-difference code bvp4c performs the three-stage Lobatto IIIa. The system of Eqs. (10–13) has been solved by a practical shooting method subject to the requirements of Eqs. (14, 15). Let’s replace $s_1=f$, $s_2=f^{'}$, $s_3=f^{'''}$, $s_4=g$, $s_5=g^{'}$, $s_6=\theta$, $s_7=\theta ^{'}$, $s_8=\phi$ and $s_9=\phi '$. We get the following first-order differential equations:

19 $$\begin{aligned} s^{'}_{1}= & {} s_{2}, \end{aligned}$$

20 $$\begin{aligned} s^{'}_{2}= & {} s_{3}, \end{aligned}$$

21 $$\begin{aligned} s^{'}_{3}= & {} \frac{1}{2} s^{2}_{2} - \frac{1}{2} s^{2}_{4} - s_1 s_3 +M s_2 -\chi _C\left( 1+\beta _c s_8 \right) s_8 - \chi _T\left( 1+\beta _T s_6 \right) s_6, \end{aligned}$$

22 $$\begin{aligned} s^{'}_{4}=\, & {} s_{5}, \end{aligned}$$

23 $$\begin{aligned} s^{'}_{5}=\, & {} s_2 s_4 - s_1 s_5 + M s_4 -\chi _C\left( 1+\beta _c s_8 \right) s_8 - \chi _T\left( 1+\beta _T s_6 \right) s_6,\end{aligned}$$

24 $$\begin{aligned} s^{'}_{6}=\, & {} s_{7}, \end{aligned}$$

25 $$\begin{aligned} s^{'}_{7}= \,& {} \frac{1}{\frac{4}{3} Rd\left( \left( \theta _w-1\right) s_{6}\right) ^{3}} \left[ -4Rd\left( \theta _w-1\right) \left( 1+\left( \theta _w-1\right) s_{6}\right) ^{2}s^{2}_{7}-Pr\left( s_1 s_7 +Nt s^{2}_7 + Nb s_7 s_9 \right) \right] , \end{aligned}$$

26 $$\begin{aligned} s^{'}_{8}=\, & {} s_{9}, \end{aligned}$$

27 $$\begin{aligned} s^{'}_{9}= & {} -Sc s_1 s_9 - \frac{Nb}{Nt} s^{'}_{7} +Sc \,\ \sigma ^{*}\left( 1+\delta s_6\right) ^n s_8 \,\ exp\left( \frac{-E}{1+\delta s_6}\right) , \end{aligned}$$

along with the boundary conditions:

28 $$\begin{aligned}{} & {} \text {at}\,\ \eta =0, \,\,\,\,\,\ s_{1}=0, \,\,\,\,\,\ s_{2}= \alpha _1 s_3, \,\,\,\,\,\ s_{4}= 1, \,\,\,\,\,\,\ s_{6}=1, \,\,\,\,\,\,\ s_{8}= 1+ \alpha _2 s_9, \,\,\,\,\ \text {and} \end{aligned}$$

29 $$\begin{aligned}{} & {} \,\,\,\text {at}\,\ ~\eta \rightarrow \infty \,\,\,\ s_2'\rightarrow 0,\,\,\,\ s_5\rightarrow 0,\,\,\,\ s_8\rightarrow 0, \,\,\,\ P\rightarrow 0. \end{aligned}$$

### Code verification

In order to confirm the validity of our most recent research, we compared the values of skin friction and Nusselt number with Yin et al.^38^ and Acharya et al.^39^, taking $Pr=6.2$ and all other parameters equal zero. An excellent level of agreement is observed with the existing literature (See Table 1)

**Table 1:** A comparison of $f'(0)$ and $-\theta ^{'}(0)$ for $Pr=6.2$.

	Yin et al.^38^	Acharya et al.^39^	Our outcomes
$f'(0)$	0.51022941	0.5102295	0.510229563
$-\theta ^{'}(0)$	0.93387285	0.9338728	0.933872847

### Ethics approval and consent to participate

The authors declare that there is no conflict with publication ethics.

## Results and discussion

**Figure 2:**
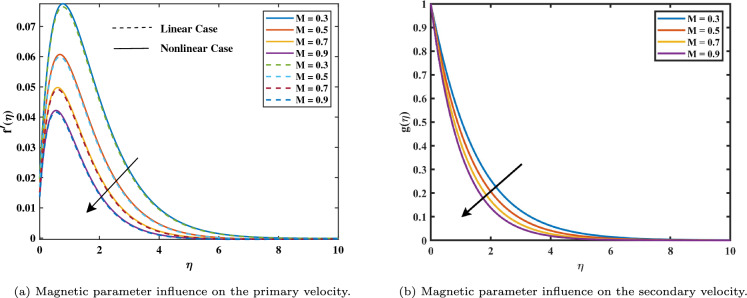
Impact of *M* on $f'$ and *g*.

**Figure 3:**
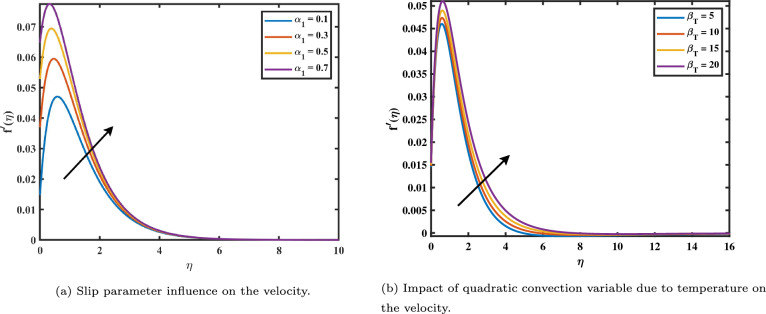
Impact of $\alpha _{1}$ and $\beta _T$ on $f'$.

**Figure 4:**
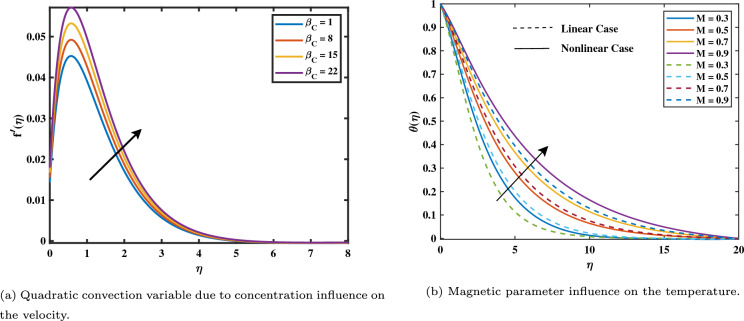
Impact of $\beta _C$ on $f'$ and *M* on $\theta$.

This research examines the nanofluid flow over a rough disc spinning with constant angular velocity using the model of Buongiorno. The system of ODEs (10–13) depending on the initial value conditions (14–15) has been investigated numerically using MATLAB with the aid of Bvp4c. For some developmental parameters such as velocity, concentration, and temperature distribution results are produced graphically. Figure 2a and b show how the velocity rate graph changes about the magnetic number $(0.3 \le M \le 0.9)$. The velocity boundary layer thickness rises as the magnetic value *M* levels rise. This is because the force known as Lorentz is induced by the presence of a magnetic transverse field, which obstructs the velocity field. As a result, the retarding force boosts along with *M*, leading to the velocity to fall. Furthermore, when the magnetic field factor increases, the boundary layer’s thickness decreases. In addition, it can be observed from Figure 2a that in the linear case, the primary velocity decreases more quickly than in the nonlinear case when the magnetic variable increases. Figure 3a shows the velocity slip graph demonstrates how the flow rate is affected by the velocity slip parameter $(0.1 \le \alpha \le 0.7)$. It has been found that the high values decrease the dimensionless velocity profile. The slip velocity rises and the fluid velocity falls as the velocity slip values rise. This is a result of the slip boundary condition occurring, the rotating disk’s speed and the flow speed nearby do not coincide. Figure 3b demonstrates the relationship between the mixed convection factors due to temperature with velocity. It shows that the velocity profile will increase with a greater value of nonlinear convection $\beta _T$. Figure 4a shows the impact of mixed convection due to the concentration $\beta _C$ on the velocity graph. It is analyzed as increasing in velocity with higher inputs of $\beta _C$.

**Figure 5:**
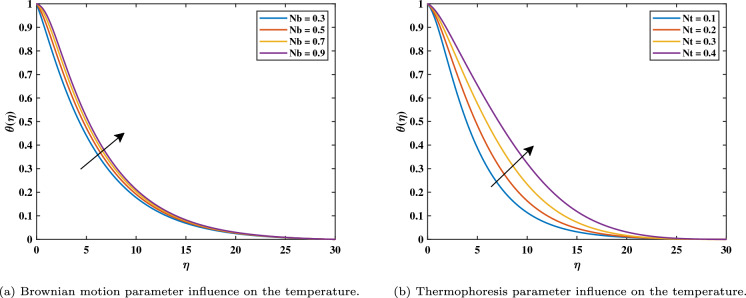
Impact of *Nb* and *Nt* on $\theta$.

**Figure 6:**
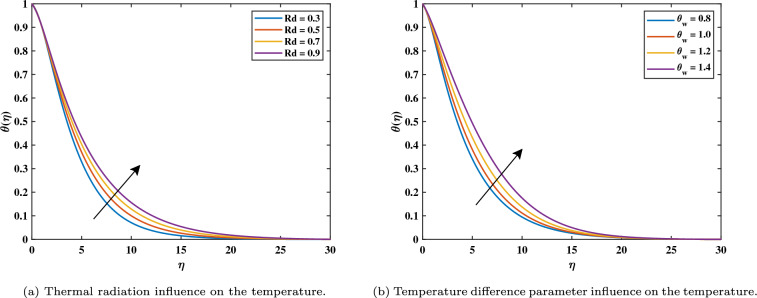
Impact of *Rd* and $\theta _w$ and $\theta$.

It has been noticed in Fig. 4b that with rising magnetic variable values $(0.3 \le M \le 0.9)$, the temperature gradient in the nonlinear form is more enhanced in the comparison linear form. The opposing Lorentz force, which exceeds the fluid movement and generates heat, is what raises the temperature. In its reaction, as the field of magnets rises, it also raises the thickness of the heat boundary layer and the concentration of the nanoparticle boundary layer. The impact of coefficients of thermophoresis (*Nt*) and Brownian motion (*Nb*) on the temperature profile is shown in Fig. 5a and b. Additionally, we see that the dimensionless heat variable and volume concentration rise when the thermophoresis factor (*Nt*) values rise $(0.1 \le Nt \le 0.4)$. This is because the heat gradient, which generates the thermophoretic effect, causes an exceptionally high rate of flow away from the rotating disk. In turn, this causes the heat boundary layer thickness to rise as (*Nt*) rises and the heat graph at the interface to reduce as both *Nt* and *Nb* variables rise. Figure 6a shows when the thermal radiation parameter increases $(0.3 \le Rd \le 0.9)$, more heat is transferred to the liquid, leading to an increased heat distribution within the boundary layer thickness. We could see from the drawing that the temperature graph behaved increasingly as the thermal radiation levels increased. Figure 6b shows how $\theta _w$ with values $(0.2 \le \theta _w \le 1.4)$ has an impact on $\theta$? Practically, the characteristic of the stronger temperature ratio raises the wall temperature above the ambient fluid temperature, resulting in a rise in the nanofluid temperature. As a result, the improvement in $\theta _w$ causes an increase in $\theta$.

**Figure 7:**
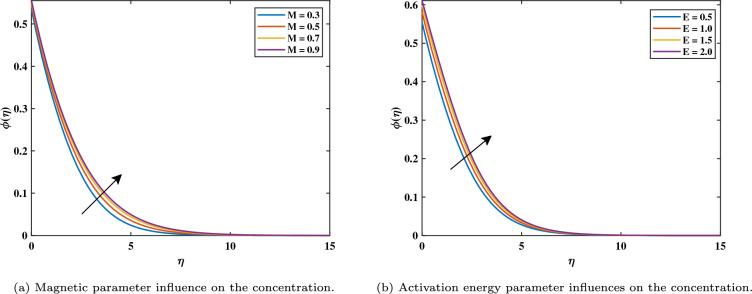
Impact of *M* and *E* on $\phi$.

**Figure 8:**
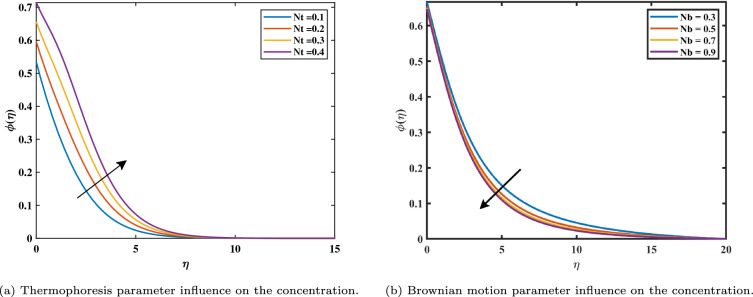
Impact of *Nt* and *Nb* on $\phi$.

**Figure 9:**
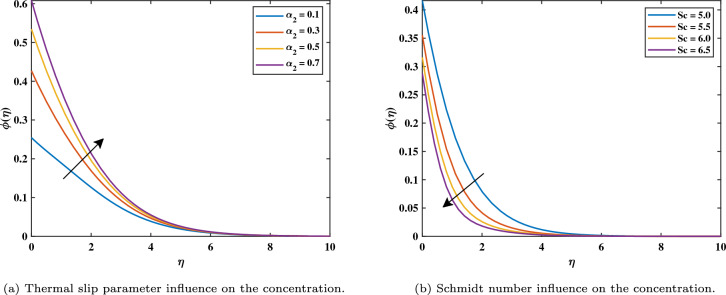
Impact of $\alpha _{2}$ and *Sc* on $\phi$.

**Figure 10:**
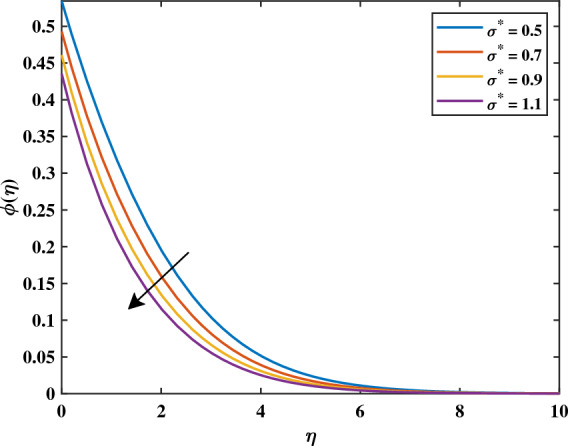
Impact of $\sigma ^{*}$ on $\phi$.

Figure 7a shows that increasing magnetic variable $(0.3 \le M \le 0.9)$ causes $\phi$ values of nanoparticle concentration increases. This is caused by the assistive Lorentz force, which reaches the movement of fluid and generates heat. Due to this, the volume fraction of nanoparticles increases, raising the concentration as a result. It can be shown in Fig. 7b that the development of activation energy $(0.5 \le E \le 2.0)$ causes $\phi$ to increase. Physically, this makes sense because of an increase in the activation energy variable. A causes the improved Arrhenius value $\left( \frac{{\hat{T}}}{T {\infty }}\right) ^n exp\left( \frac{-E^{*}}{K T}\right)$ to decrease, which subsequently boosts the chemical process. It can be noticed in Fig. 8a that with the increasing value of *Nt* the gradient of the concentration graph rises. This is because the particles of nanofluid migrate from an area of greater heat to an area of less heat as a result of the thermophoresis’s effect versus the gradient of temperature. Figure 8b exhibits a declining tendency due to the concentration falling towards the variable Nb. This is due to the erratic motion of the particles of fluid. Figure 9a shows that the concentration increases with the enhancement of the thermal slip variable. The Schmidt number *Sc* on the field of concentration is investigated in Fig. 9b. It is noticed that the profile rising value of $(5.0 \le Sc \le 6.5)$ concentration falls because mass diffusion is reduced. The graph in Fig. 10a illustrates how the chemical reaction parameter $\sigma ^{*}$ affects the concentration slope. It should be noted that a lower concentration is generated by a higher $\sigma ^{*}$ level $(0.5 \le \sigma ^{*} \le 1.1)$. Physically, concentration collapses as a result of an increase in the species’ reaction velocity, which improves the approximation of chemical reaction qualities.

Tables 2 and 3 provide the quantitative results of the skin friction coefficient and Nusselt number for various parameter values. Table 2 shows the skin friction coefficient enhanced with the increasing values of the magnetic variable. The velocity distribution next to the boundary can be impacted by the Lorentz force brought on by the magnetic field, which could result in a rise in skin friction. Temperature-related increases in the mixed convection variable point to a greater impact of buoyancy forces brought on by temperature and concentration gradients. This indicates a greater slip between the fluid and the solid surface and has the potential to change the flow dynamics close to the boundary. Therefore, it states to the rise in skin friction. Table 3 shows the Nusselt number decreases in both cases (linear and nonlinear) with the increasing values of the thermophoresis coefficient and $\theta _w$. It results from increased thermophoretic effects that influence heat transfer through convection. With the increase in the magnetic variable and thermal radiation, the magnitude of the Nusselt number in linear and nonlinear cases is also reduced. A higher Brownian movement in the fluid tends to distribute the nanoparticles better. Concentrated nanoparticles may decrease the transfer of heat effectiveness and impede fluid flow, which reduces the value of the Nusselt number in both cases.

**Table 2:** Numeric outcomes of skin friction coefficient versus different inputs of parameters.

*M*	$\beta _{T}$	$\beta _{C}$	$\alpha _{1}$	$Re^{0.5}C_{f}$
0.4	2.0	0.1	0.5	0.7442
0.8				0.9465
1.2				1.1272
1.0	0.2			1.0462
	0.4			1.0473
	0.6			1.0477
	0.5	0.3		1.0663
		0.6		1.0976
		0.9		1.1308
		0.1	0.2	1.0437
			0.5	1.0525
			0.8	1.0586

**Table 3:** Numeric outcomes of Nusselt number versus different inputs of parameters.

*M*	*Rd*	*Nt*	*Nb*	$\theta _{w}$	$Re^{-0.5}Nu$	$Re^{-0.5}Nu$
					Linear case	Non-linear case
0.4	0.5	0.1	0.1	0.9	0.3750	0.3957
0.8					0.2282	0.2489
1.2					0.1536	0.1714
1.0	0.6				0.1970	0.2172
	1.0				0.2433	0.2650
	1.4				0.2849	0.3074
	0.5	0.2			0.1602	0.1764
		0.3			0.1399	0.1538
		0.4			0.1234	0.1350
		0.1	0.2		0.2482	0.2615
			0.5		0.1844	0.2040
			0.8		0.1359	0.1584
			0.1	0.8	0.1903	0.2113
				1.1	0.1683	0.1845
				1.4	0.1382	0.1494

## Conclusions

This paper describes the flow of nanofluids with MHD liner and nonlinear mixed convection, nonlinear thermal radiation, activation energy, and slip conditions effects over a rough rotating disk. The physical problem is made up of the nonlinear PDEs system. A suitable similarity transformation is used to convert these PDEs into ODEs. To handle these ODEs, a numerical approach known as Bvp4c built-in Matlab is used. There are some findings are there.

- It is discovered that when the value of the magnetic parameter increases, the primary and secondary velocity components both decrease.
- Analysis reveals that the principal linear flow of the fluid decreases more quickly than the nonlinear flow with the increased value of *M*.
- As the velocity slip coefficients increased, the primary flow rate also increased.
- The investigation shows that when the magnetic value increases, the nonlinear temperatures rise more quickly than the linear temperature profile.
- The temperature profiles enhanced for higher values of the magnetic variable, the thermophoresis variable, the Brownian motion variable, and the thermal radiation variable.
- For higher inputs of the Schmidt number, chemical reaction value, and Brownian motion parameter, the concentration profiles declined.
- The increasing value of thermal slip parameter, activation energy, and magnetic parameter, the profile of concentration inclined.
- The physical parameters such as skin friction coefficient increase and the Nusselt number decreases with the increase of magnetic variable.
- The magnitude of the Nusselt number decreases with the rising alue of *Nt* and *Nb*.
- The magnitude of skin friction rises with the increasing value of mixed convection variable due to temperature and concentration.

To fulfill the goal of this research, the following research questions have to be addressed:

- Based on the Buongiorno model, what effects does change the concentration of nanoparticles in the nanofluid possess on its thermal transmit properties over a rotating disk?
- What is the effect of linear/nonlinear mixed convection and magnetic parameters on velocities, temperature, and concentration profiles?
- How does the Buongiorno model forecast the thermal transmit efficiency of nanofluids across rotating disks compared to that of conventional fluids without nanoparticles?
- How does the boundary layer flow of nanofluid significantly change when physical variables like internal heating and thermal radiation are present?
- What effects do the thermophoresis and Brownian motion of small particles have on the transmission of heat?
- Examine the impact of the energy of activation, Schmidt number, and chemical reaction parameters on the fluid’s concentration of nanoparticle properties.

## Data Availability

The data that support the findings of this study are available from the corresponding author upon reasonable request.

## List of symbols

$\rho _f$: Base fluid density (kg/m$^3$)
${C}_\infty$: Concentration away from the surface
$\bar{D_T}$: Thermophoretic diffusion coefficient (m$^2$/s)
${C}_\infty$: Concentration away from the surface
*M*: Magnetic parameter
*g*: Dimensionless secondary velocity
${\bar{\tau }}$: Heat capacity of nanoparticle substance (J/K)
$\rho {Cp}$: Specific heat capacity (JKg$^1$K$^1$)
$B_o$: Magnetic field variable $(\Omega ^{{ - 1/2}} {\text{m}}^{{ - 1}} {\text{s}}^{{ - 1/2}} {\text{kg}}^{{1/2}} )$
$\sigma ^{*}$: Stefan-Boltzmann constant force ${\text{K}}$
$\lambda _3$,$\lambda _4$: Mixed convection due to temperature $\lambda _1$,$\lambda _2$ ] Mixed convection due to concentration
$\chi _T$: Nonlinear mixed convection due to temepareture
$\chi _C$: Nonlinear mixed convection due to concentration
${T}_{\infty }$: Temperature away from the surface (K)
$${\bar{\delta }}$$: Temperature difference (K)
$$\eta$$: Dimensionless variable
$\bar{L_1}$: Wall slip coefficient
$\alpha _1$: Velocity slip coefficient (m/s)
$\nu _f$: Kinematic viscosity of nanofluid $({\text{m}}^{2} /{\text{s}})$
$K_R$: Chemical reaction factor (1/s)
$\bar{D_B}$: Brownian diffusion variable $({\text{m}}^{2} /{\text{s}})$
$${\bar{Nu}}_{x}$$: Nusselt number
*Sc*: Schimit number
(*U*, *V*, *W*): Velocity components (m/s)
*Pr*: Prandtl number
*E*: Activation energy (J)
$$g^{*}$$: Gravitational acceleration $$({\text{m}}^{2} /{\text{s}})$$
$\sigma _1$: Rate of chemical reaction
$$\alpha _f$$: Thermal diffusivity $$({\text{m}}^{2} /{\text{s}})$$
$K_T$: Chemical reaction due to tempreture
$$T_\theta$$: Dimensionless temperature (K)
${\bar{T}}$: Non-dimensional Temperature (*K*)
*Rd*: Radiation parameter
*C*: Concentration parameter $$({\text{Mol}}\;{\text{L}}^{{ - 1}} )$$
$f'$: Dimensionless primary velocity
$$\phi$$: Dimensionless concentration
$\bar{L_2}$: Temperature jump coefficient
$\alpha _2$: Thermal slip coefficient $({\text{K}}^{{ - 1}} )$
$\theta _w$: Ratio of temperature difference

## Future directions

This work can be extended for the modified Buongiorno model with different non-Newtonian fluids such as Casson, Williamson, and Ellis fluids.
